# A harder problem of consciousness: reflections on a 50-year quest for the alchemy of qualia

**DOI:** 10.3389/fpsyg.2025.1592628

**Published:** 2025-05-14

**Authors:** Juan D. Gomez

**Affiliations:** Kettering University Online (KUO), Kettering University, Flint, MI, United States

**Keywords:** qualia, consciousness, space, perception, light, IIT, artificial intelligence

## 1 Introduction

Consciousness is a fundamental aspect of human nature, yet it remains one of the most elusive scientific and philosophical challenges. Explaining what it means to have an experience—what philosophers call *qualia*—is a conundrum that has perplexed thinkers for centuries. Arguably, Thomas Nagel helped bring the concept of qualia into mainstream philosophical discourse over 50 years ago with his seminal paper from 1974: *What Is It Like to Be a Bat?* (Nagel, 1974), which articulated the difficulty of explaining subjective experience in objective terms. More recently, the question of why and how humans and other organisms have qualia has been framed as the *Hard Problem of Consciousness*, a term coined by David Chalmers in 1995 (Chalmers, 1995).

Unambiguously, the *Hard Problem of Consciousness* concerns the question of why neural activity is accompanied by subjective experience—why there is a qualitative aspect to cognition, rather than brain processes in the dark, i.e., simply unfolding in a purely mechanistic, unconscious manner (Chalmers, 1995). One of the most classic examples of *qualia* is the subjective perception of color—commonly illustrated by the “redness” of red:

*When I observe a red apple, reflected light from its surface travels through space, enters my eyes, and is focused onto my retina, where it stimulates specific photoreceptors. From this point onward, the information is converted into electrical signals that propagate through the optic pathways via the cranial nerves and into the brain—a structure weighing approximately three pounds, containing nearly 86 billion neurons and over 500 trillion synapses. In complete silence and total darkness, the sensation of redness emerges within my conscious awareness, appearing to be projected back into space, clothing the apple as though it inherently possesses that color*.

Notice that while the color red can be fully described in terms of objective physics—such as its wavelength of approximately 650 nanometers—the subjective experience of *redness* (i.e., *qualia*) remains inherently private to the observer (Nagel, 1974). This qualitative aspect of cognition is inaccessible to others, each of whom possesses their own subjective experiences; and it is especially unattainable for someone blind from birth, even if they have a complete theoretical understanding of color perception (Dennett, 1991).

Examples like the one above, along with others of a similar nature—such as the *qualia* of pain or sound—have centered the debate around the ***alchemy of qualia***: *how does the water of the brain transform into the wine of consciousness?* Yet this question is asked within a descriptive framework that we seldom interrogate—as if it were fully understood and epistemologically sound. Challenging that view, I opine that the *Hard Problem of Consciousness* may have been framed on questionable grounds from the beginning—overlooking the possibility that the mystery arises even prior to the explanatory gap we commonly refer to as the alchemy of qualia. For the universe is not only stranger than we think—but stranger than we can think (Searle, 1998).

## 2 Are we looking for the right qualia?

Discussions on consciousness have traditionally focused on the emergence of qualia within the brain's neural architecture (Nagel, 1974)—an explanatory gap often referred to as the *alchemy of qualia*. For instance, we have long questioned how the subjective sensation of *redness* arises from electrochemical activity in neural networks, triggered by electromagnetic waves propagating through space. This perspective, however, presupposes an implicit ontological framework in which space itself is taken as an objective, independent substrate within which consciousness unfolds. Yet, a crucial question is: *through what space does the red-light wave actually propagate?*

Space, often mistaken for a neutral stage, is itself a perceptual construct within consciousness—analogous to the qualia of *redness* (Merleau-Ponty, 1962). In explaining *redness*, we are not merely addressing an isolated percept but relying on another percept—*extendedness*—to ground our explanation. Consequently, scientific frameworks for studying consciousness, rooted in empirical observation, are inherently built upon perceptual categories that are themselves artifacts of the very mind they seek to explain (Nagel, 1974). This, of course, introduces a form of circularity that challenges the adequacy of our current paradigms and explanatory models.

If space—the supposed absolute container of all things—is removed from the explanandum, the phenomenon of redness becomes intractable even before we reach the so-called alchemy gap: where, in the “outside world,” do the brain and light waves converge to generate consciousness —of red—, if that very “outside world”—i.e., space—is already contained within consciousness itself? We often believe the mystery lies solely in the explanatory gap—the alchemy of qualia—but that's because we take **space** for granted, using it as the stage on which the physical precursors of consciousness are placed. Yet to do so, we must first elucidate **space itself** within a similar third-person explanatory framework (Graziano and Cooke, 2006). If space happens to be another **quale**, then we must ask—before even asking for its alchemy—: *what is its precursor?* Just as light waves are said to precede redness, what precedes space? This new view may expose deeper epistemological tensions.

Are we merely navigating the recursive loops of our own perception, endlessly chasing shadows cast by the mind upon itself, mistaking the map for the territory? Would a first-person phenomenological approach—rather than a third-person reductionist framework—offer a more viable means of inquiry? Or does the very act of theorizing about consciousness necessitate the assumption of an objective world, thereby inextricably tying us to the paradox of self-reference? The question persists: is the apparent objectivity of the physical world merely a high-order cognitive construction, a heuristic convenience rather than a fundamental reality?

The effort to explain space—let alone time—in order to securely position the elements needed to merely *describe* the Hard Problem of Consciousness, the so-called alchemy of qualia, is what I refer to as the *Harder Problem* of Consciousness.

This problem extends beyond neuroscience and philosophy into epistemology itself, demanding a radical reconsideration of the very conditions that make knowledge possible. If consciousness underlies all epistemic structures, then the distinction between subject and object—between perception and reality—may not be an absolute metaphysical divide but rather an artifact of cognition itself.

## 3 The problem of space perception

Neuroscientific evidence increasingly suggests that our perception of space may diverge significantly from its underlying nature—implying that the seemingly external structure in which we passively exist is, in fact, an active construct generated within the brain (O'Keefe and Dostrovsky, 1971). The hippocampal-entorhinal system, for instance, constructs an internalized spatial framework through the activity of place cells, grid cells, and head direction cells (Hafting et al., 2005; Taube et al., 1990). Studies on rodents and humans have shown that these neurons create a dynamic, topological representation of space, independent of any direct sensory input. Furthermore, damage to these structures leads not only to disorientation but also to a disruption in spatial continuity, suggesting that our experience of extendedness is not a direct reflection of an external world but an internally generated model (O'Keefe and Dostrovsky, 1971).

Empirical evidence further supports this view. Unlike ultraviolet light—which does not produce a corresponding quale and thus escapes subjective representation—we do experience space. Yet the subjective experience of space varies widely across species with different sensory modalities, as observed in bats (Ulanovsky and Moss, 2007). This suggests that the space we perceive is not a direct reflection of objective reality—if any—, but rather a phenomenal construct shaped by the mind. Thus, the question deepens: just as electromagnetic waves elicit *redness*, what gives rise to *extendedness*? Without a foundational understanding of this, any discussion of qualia—including the *Hard Problem of Consciousness*—remains secondary and perhaps even futile.

### 3.1 Hypothetical scenario for a misformulated hard problem of consciousness

There may be numerous scenarios where our current explanandum of qualia proves inadequate or entirely impractical, all contingent on the nature, structure, and behavior of physical space *outside perception* (Levine, 1983). The physical correlate of our qualia of space may be as fundamentally distinct from what *extendedness feels like* as a digital folder on a screen is from its correlates (transistors). If our cognitive architecture is intrinsically constrained by the very phenomenon we seek to explain, then conceiving such scenarios may require an epistemic leap—one that, in theory, transcends the perceptual limits imposed by our cognition. A way harder problem, indeed.

In what follows, let us explore one hypothetical—yet plausible—scenario among countless possible alternatives regarding the nature of space, distinct from the one we unquestionably assume.

#### 3.1.1 The reversal of space and light

We know as an empirical fact that no time elapses for a photon from its emission to its absorption—it experiences no passage of time (Einstein, 1905; Wheeler, 1990). Yet this raises an unsettling question: how can we conceive of a region of space, however small or large, for which travel occurs instantly? A hypothetical explanation to this fact could be that *space itself comes into being only when the photon reaches the eye of the observer*. If this were the case—just hypothesizing—, it would imply that space exists *within* light, rather than light *within* space (Penrose, 2004). I use this hypothesis here just as an open epistemic challenge, one that cannot be falsified until we have an unequivocal understanding of our perception of spatiality.

Let us consider the following points that support this hypothetical—not factual—scenario and challenge our conventional assumptions:

- **Constancy of light's speed:** unlike any other motion, the speed of light remains invariant for all observers, regardless of their velocity, suggesting it fundamentally defines space itself (Einstein, 1905; Minkowski, 1908).
- **Light defines spatial geometry:** a light beam's trajectory determines a “straight line” in space. If space were an independent medium, why would light's motion dictate spatial relationships? (Minkowski, 1908; Wheeler, 1990).
- **No physical medium:** sound needs air, and water waves need liquid, but light propagates in a vacuum. The absence of an ether suggests space is not an independent entity but a structure imposed by light (Michelson and Morley, 1887).
- **Speed of light as a universal limit:** all massive objects are constrained by c. If space were independent, why would it impose this strict speed limit? (Einstein, 1905; Ellis and Uzan, 2005).
- **Time dilation:** as an object nears c, time slows; at c, time stops. If light simply moved through space, why would its motion alter time itself? (Einstein, 1905; Wheeler, 1990).
- **Length contraction:** objects shrink in their direction of motion near c, suggesting spatial extension depends on an observer's velocity relative to light (Minkowski, 1908).
- **Light's unique behavior:** unlike ordinary objects, light's wave-particle duality, massless momentum, and absolute speed challenge classical mechanics (Feynman, 1965).
- **Space as an emergent property:** if space emerged from light, its constraints—such as the speed limit, time dilation, and causality—would make more sense, rather than space defining light's behavior (Penrose, 2004; Misner et al., 1973).
- **Light's unmatched properties:** light always moves at c, never rests, exists outside time, and maintains the same speed for all observers, defying classical spatial laws (Ellis and Uzan, 2005).

#### 3.1.2 A paradigm shift in understanding space

If light were merely an entity within space, its unique properties would appear anomalous. However, if space emerges from light, these constraints become fundamental characteristics. The limit *c* may not just govern motion but define space itself.

If the above were true—as may be the case with many other possible explanations for space—then consciousness has been mistakenly conceived as something that exists *within* space. Even more radically, it would imply that the physical universe is not a structure in which we exist, but rather the structure of consciousness itself. Consequently, there would be no existence outside of consciousness, because “outside”—extendedness—belongs within it.

In this context, the issue with the *Hard Problem of Consciousness* is that, in attempting to explain *redness*, it relies on a photon crossing the room, treating space as a conceptual given. However, this hypothetical reconceptualization of space, light, and consciousness fundamentally challenges this framework, revealing at least one—among many—scenarios in which an even *Harder Problem of Consciousness* emerges.

## 4 Discussion and conclusion on the future of the qualia adventure

Integrated Information Theory (**IIT**) is the only leading framework that explicitly incorporates space into its model of consciousness (Tononi, 2004). However, IIT addresses *phenomenal* rather than *physical* space (Haun and Tononi, 2019). This is consistent with its fundamental reversal of the *Hard Problem*: rather than explaining how the brain generates consciousness, IIT begins with consciousness and determines what physical systems could instantiate it. Consequently, whether physical space exists independently or not becomes irrelevant.

Yet, this dismissal raises profound questions: if, within its *extendedness*, the universe contains all we know, but is itself contained within consciousness as a **phenomenal** structure, where can IIT-based conscious **physical** systems be found? Is consciousness all there is? IIT may provide a solution to consciousness, but it does so within a universe so fundamentally alien that comprehending it leads to an even *Harder Problem*.

Moreover, artificial intelligence (AI), in its current form, is fundamentally incapable of generating qualia. Large language models process information to simulate intelligence through linguistic structures, yet they make no attempt to instantiate subjective experience. Moreover, artificial neural networks, as their name implies, do not physically exist—they are mathematical simulations rather than tangible systems. This distinction is crucial: since artificial networks do not *occupy* space, they cannot *perceive* space. If, as I argue, spatial perception is a prerequisite for qualia, then AI—lacking spatial existence—remains inherently incapable of consciousness.

Some may argue that space lacks a physical correlate, as it arises from internal brain processes like thought. Others, however, contend that unlike thoughts, space is perceived universally, suggesting an external cause for our common experience of it. In either case, the problem of explaining consciousness only grows harder.

Finally, this reassessment of the *Hard Problem of Consciousness* challenges conventional notions of what it truly means to be a vortex of experience in an utterly unknown universe. It raises deeper existential questions about the nature of consciousness, the fundamental structure of reality, and the fabric of space and time—forcing us to reconsider whether we are genuinely on a path toward a *science of the alchemy of qualia*.
